# 单孔与三孔胸腔镜治疗非小细胞肺癌患者近期结果对比分析

**DOI:** 10.3779/j.issn.1009-3419.2018.12.03

**Published:** 2018-12-20

**Authors:** 高祥 王, 燃 熊, 汉然 吴, 广文 徐, 彩伟 李, 效辉 孙, 世斌 徐, 美青 徐, 明然 解

**Affiliations:** 230001 合肥，安徽医科大学附属省立医院胸外科 Department of Thoracic Surgery, Anhui Provincial Hospital Affiliated to Anhui Medical University, Hefei 230001, China

**Keywords:** 肺肿瘤, 电视胸腔镜, 单孔, 疗效, Lung neoplasms, Video-assisted thoracic surgery, Uniportal, Efficacy

## Abstract

**背景与目的:**

目前多孔胸腔镜肺部手术治疗肺部疾病的优势研究报道较多，但单孔和多孔胸腔镜（video-assisted thoracic surgery, VATS）之间的比较报道较少。本研究旨在评估单孔与三孔VATS两种手术方式对非小细胞肺癌（non-small cell lung cancer, NSCLC）患者术后近期疗效的比较。

**方法:**

回顾性分析266例行单孔或三孔VATS肺癌根治术的患者，比较两组患者临床病理特征、围手术期资料和短期生活质量。

**结果:**

两组患者临床病理特征无统计学差异（*P* > 0.05）；相较三孔组，单孔组术中出血量和术后引流量更少，术后胸管留置时间和术后住院时间更短（*P* < 0.05），两组患者淋巴结清扫数目和站数无统计学差异（*P* > 0.05）。单孔组术后疼痛得分明显低于三孔组（*P* < 0.05）。单孔组总并发症、肺部并发症发生率低于三孔组，无统计学意义（*P* > 0.05）。

**结论:**

单孔VATS作为一种更加微创的手术，治疗可切除NSCLC安全有效，能够达到甚至优于三孔VATS手术效果。

## Abstract (en)

**Background and objective:**

Currently, there are many reports on the advantages of three portal video-assisted thoracic surgery (VATS) in the treatment of lung cancer, but there are few reports on the comparison between uniportal and three portal video-assisted thoracic surgery. In this study, we aimed to evaluate the recent curative effect of the postoperative short-term quality of life between uniportal and three portal video-assisted thoracic surgery for non-small cell lung cancer.

**Methods:**

We retrospectively evaluated 266 patients with NSCLC who underwent intended VATS lobectomy by a single surgical team in our ward between January 2016 and August 2017. The general clinical date, perioperative data and short-term life quality were individually compared and analyzed between the two groups.

**Results:**

The two groups were similar in terms of clinicopathological features, total number of dissected lymph nodes and nodal stations, postoperative complications and pulmonary complications (*P* > 0.05). Compared with three portal VATS, the intraoperative blood loss, chest tube duration, postoperative thoracic drainage, length of stay and NRS score were significantly decreased in uniportal VATS, with significant differences (*P* < 0.05).

**Conclusion:**

As a more minimally invasive surgery, uniportal VATS can be safely and effectively performed for resectable lung cancer, which would achieve even better operation curative effect than three portal VATS.

肺癌发病率和死亡率在全世界范围内一直居于所有恶性肿瘤之首^[1]^。以手术为主的综合治疗是可切除非小细胞肺癌（non-small cell lung cancer, NSCLC）的主要治疗手段。相对于传统的开胸手术，胸腔镜微创手术具有创伤小、恢复快等优点，其疗效已经被广泛认可^[2]^。2006年美国国家综合癌症网络（National Comprehensive Cancer Network, NCCN）指南中将胸腔镜（video-assisted thoracic surgery, VATS）肺叶切除术作为肺癌外科治疗的标准术式^[3]^。目前国内外大多数医学中心采用多孔胸腔镜（二孔、三孔和四孔）作为肺癌微创手术入路^[4]^。2004年Rocco等^[5]^报道了首例单孔VATS肺楔形切除，2011年Gonzalez等^[6]^首次报道了单孔VATS肺叶切除术和系统淋巴结清扫术，随后在全世界范围内掀起了单孔胸腔镜手术的研究热潮。我科自2014年9月开始实施单孔胸腔镜肺部手术，由最初的楔形切除逐步过渡到肺叶、肺段切除以及全肺和袖式切除手术。多孔胸腔镜肺部手术治疗肺部疾病的优势研究报道较多^[2, 4]^，但单孔VATS和多孔VATS之间的比较报道较少。本研究旨在探讨单孔VATS手术技术，并比较该术式和传统三孔VATS手术对NSCLC患者术后近期结果是否有差异。现报道如下。

## 1 资料与方法

### 1.1 一般资料

回顾性分析安徽医科大学附属省立医院胸外科2016年1月-2017年8月期间接受单孔或三孔胸腔镜肺叶切除术的NSCLC患者266例，其中男性151例，女性115例，平均年龄（61.84±9.84）岁。按照手术方式不同分为两组，其中接受单孔胸腔镜手术（单孔组）153例，接受三孔胸腔镜手术（三孔组）113例。

患者的术前检查包括：血常规、生化、凝血象、免疫组化、心电图、超声心动图、肺功能、气管镜检查，同时行胸部平扫+增强计算机断层扫描（computed tomography, CT）明确病变性质，行腹部+双侧肾上腺彩超、头颅磁共振成像（magnetic resonance imaging, MRI）平扫+增强、骨扫描检查以排除远处转移，部分患者行正电子发射计算机断层显像（positron emission tomography-CT, PET-CT）。所有患者的肿瘤分期采用第8版肿瘤-淋巴结-转移（tumor-node-metastasis, TNM）分期系统。术后采用Clavien-Dindo外科并发症分级标准评价并发症发生情况，将Clavien-Dindo 1级-2级并发症归为微小并发症，将Clavien-Dindo 3级-5级并发症归为重大并发症。

### 1.2 纳入与排除标准

纳入标准：①病理证实为非小细胞肺癌；②手术方式为单孔或三孔胸腔镜肺叶切除手术，接受系统性肺门及纵隔淋巴结清扫；③术前分期为cT_1-2_N_0-1_M_0_；④R0切除。排除标准：①双侧同期手术的患者；②临床资料不完整。

### 1.3 手术方法

单孔组：患者静脉吸入复合全麻，健侧卧位，单肺通气。取患侧腋前线和腋中线第4或第5肋间3.0 cm小切口，切口放置切口保护套。先置入胸腔镜探查胸腔有无粘连及播散结节，再确定病灶位置及肺门解剖情况。对于周围型病灶，先行楔形切除，术中冰冻明确病理为肺癌后再行肺叶切除+纵隔淋巴结清扫术。根据叶裂发育情况选择游离顺序，若肺裂发育良好优先处理动脉，若叶裂发育不佳，可采用“隧道式”或“单向式”方法切除。5 mm以下血管采用双重结扎后超声刀离断，5 mm以上血管、气管和发育不良的肺裂采用内镜下直线切割缝合器离断。右侧肺癌常规探查并清扫第2R、3A、3P、4R、7、8、9、10组淋巴结及肺内淋巴结，左侧肺癌常规探查并清扫第4L、5、6、7、8、9、10组淋巴结及肺内淋巴结。

三孔组：麻醉方式同体位同单孔组，取腋前线第4或第5肋间3 cm切口为主操作孔，腋中线第7或第8肋间1.0 cm切口为观察孔，腋后线第7肋间1.5 cm切口为辅助操作孔。术中助手经辅助操作孔协助暴露术野，需要时可经辅助操作孔置入切割缝合器。肺叶切除及淋巴结清扫同单孔组。

### 1.4 观察指标

比较分析两组患者的临床病理资料、围手术期资料、术后数字评分法（numeric rating scale, NRS）评分、淋巴结清扫的站数和枚数、术后并发症发生率。

### 1.5 统计学方法

采用SPSS 24.0统计学软件对数据进行分析。计量资料采用均数±标准差（Mean±SD）表示，计量资料比较采用*t*检验，计数资料比较采用*χ*^2^检验。*P* < 0.05为差异有统计学意义。

## 2 结果

### 2.1 临床资料比较

两组患者在年龄、性别、pTNM分期、病理类型和分化程度方面差异均无统计学意义（*P* > 0.05），见表 1。

**1:** 两组术前临床资料比较 Comparisons of clinical characteristics between uniportal group and three portal group

Characteristics	Uniportal group (*n*=153)	Three portal group (*n*=113)	*t*/*χ*^2^	*P*
Gender			1.477	0.224
Male	82	69		
Female	71	44		
Age (yr)	61.52±9.70	62.27±10.08	0.222	0.536
Pathological types			1.247	0.536
Squamous cell carcinoma	26	25		
Adenocarcinoma	115	81		
Others	12	7		
Tumor location			1.048	0.902
RUL	54	36		
RML	15	9		
RLL	27	21		
LUL	35	31		
LLL	22	16		
Differentiation			4.340	0.114
HD	10	3		
MD	53	51		
LD	90	59		
TNM stage			5.556	0.062
Ⅰ	115	72		
Ⅱ	14	10		
Ⅲ	24	31		
TNM: tumor-node-metastasis; HD: highly differentiated; MD: moderately differentiated; LD: lowly differentiated.				

### 2.2 围手术期资料比较

单孔组的术中出血量、术后引流量、术后胸管留置时间和术后住院时间方面较三孔组低，差异有统计学意义（*P* < 0.05）。单孔组与三孔组在手术时间上无统计学差异（*P* > 0.05）。两组患者在淋巴结清扫的站数和枚数方面差异无统计学意义（*P* > 0.05）。单孔组24 h和72 h术后NRS评分低于三孔组，有统计学差异（*P* < 0.05），见表 2。

**2:** 两组患者围手术期比较（Mean±SD） Comparisons of perioperative parameters between uniportal and three portal group (Mean±SD)

Index	Uniportal group (*n*=153)	Three portal group (*n*=113)	*χ*^2^	*P*
Intraoperative blood loss (mL)	73.01±32.87	89.73±48.65	7.589	0.002
Postoperative hospital stay (d)	7.83±3.07	9.44±4.81	12.877	0.002
Operation time (min)	193.05±59.99	212.22±77.53	9.728	0.082
Postoperative thoracic drainage (mL)	876.80±668.01	1, 141.42±746.83	1.064	0.003
Chest tube duration (d)	6.35±3.40	7.54±3.95	3.956	0.010
The number of total lymph nodes dissected	16.46±8.33	16.61±8.02	0.237	0.880
The stations of the total lymph nodes dissected	5.11±1.71	4.93±1.78	0.022	0.401
24 h postoperative pain NRS score	4.84±0.83	5.12±1.05	4.282	0.017
72 h postoperative pain NRS score	3.29±1.04	3.70±1.16	2.698	0.003
NRS: numeric rating scale.				

### 2.3 术后并发症

全组共89例出现术后并发症，其中单孔组45例（29.4%），三孔组44例（38.9%），两组比较无统计学差异（*P*=0.104）。另外，单孔组肺部并发症发生率低于三孔组，差异无统计学意义（22.2% *vs* 28.3%, *P*=0.255）。进一步分层研究发现，单孔组在肺漏气、肺部感染、肺不张方面均低于三孔组，均无统计学差异，见表 3。

**3:** 两组患者术后并发症发生率比较 Comparisons of postoperative complications between uniportal and three portal group

Index	Uniportal group (*n*=153)	Three portal group (*n*=113)	*χ*^2^	*P*
Minor complications（Clavien-Dindo grade 1-2）				
Pulmonary leakage	19 (12.4%)	17 (15.0%)	0.383	0.536
Pulmonary infection	6 (3.9%)	4 (3.5%)	0.000	1.000
Atelectasis	4 (2.6%)	6 (5.3%)	0.666	0.414
Incisional infection	2 (1.3%)	4 (3.5%)	0.631	0.427
Arrhythmia	6 (3.9%)	3 (2.7%)	0.041	0.840
Major complications (Clavien-Dindo grade 3-5)				
Pulmonary infection	4(2.6%)	3 (2.7%)	0.000	1.000
Chylothorax	1 (0.7%)	2 (1.8%)	0.070	0.791
Bronchial stump fistula	1 (0.7%)	2 (1.8%)	0.070	0.791
Reoperation	2 (1.3%)	3 (2.7%)	0.118	0.731
Postoperativecomplications rate	45 (29.4%)	44 (38.9%)	2.649	0.104
Pulmonary complications rate	34 (22.2%)	32 (28.3%)	1.295	0.255

## 3 讨论

三孔VATS加系统纵隔淋巴结清扫在NSCLC患者的手术治疗中与传统开胸手术效果相当，且具有创伤小、恢复快、淋巴结清扫彻底和并发症少等优点，是治疗NSCLC的有效办法^[7]^。其主要优势在于，三孔VATS全景视野效果更佳，且三个手术切口器械基本相互不构成干扰，可完成大多数胸外科常规手术。单孔VATS在不影响手术范围的前提下，将手术切口减少到1个，最大限度地减少了手术的创伤，已成为近年来胸腔镜手术发展的主要趋势之一。目前在国内少数大的医学中心，单孔胸腔镜手术已成为肺部手术的主流术式^[8, 9]^。从理论上讲，单孔胸腔镜手术存在以下优势：①最大限度地减少了肋间神经的损伤，减轻了患者术后切口疼痛；②使用软性切口保护套，避免了戳卡和副操作孔器械使用对切口的反复挤压和摩擦导致的切口出血；③镜身与操作器械同孔进入，虽不能达到三孔VATS的全景视野，但单孔VATS视野更接近于传统开放手术视野，利于术者完成学习曲线，甚至对部分位置的胸腔粘连松解更具优势；④更为美观的手术切口，患者主观上更易接受，提高了患者术后的生活质量。本研究发现，相对于三孔胸腔镜手术，单孔胸腔镜肺部手术可达到同样的淋巴结清扫范围，不增加手术时间和术后并发症发生率。同时，在术中出血量、术后疼痛和术后住院时间方面具有明显优势。进一步论证了单孔胸腔镜手术在肺癌微创外科治疗中的地位和优势。

本研究发现，单孔VATS在术中出血量、术后引流量和术后住院时间方面较三孔VATS具有明显优势。韦海涛等^[10]^通过对50例行单孔或三孔胸腔镜手术的NSCLC患者的临床资料进行分析后发现，单孔组在术中出血量、术后引流总量、术后胸管留置时间方面明显优于三孔组。Wang等^[11]^通过50例行单孔VATS和183例行三孔VATS肺癌患者的比较发现，单孔组较三孔组术中出血量更少、术后住院时间更短和术后引流量更少。我们认为其主要原因在于：①手术切口的减少导致术中切口出血和术后局部的水肿渗出减少，进而减少了术后引流量和胸管留置时间；②三孔组由于戳卡和副操作孔器械使用对切口的反复挤压和摩擦导致切口反复出血和止血，进而增加了术中出血量和术后引流量；③单孔手术视野更为接近开放视野，更符合手术者习惯，使镜下操作更为精细，更大限度减少了手术的误伤，进一步减少了术中出血和术后引流。

术后切口麻木疼痛与患者术后近期生活质量显著相关，术后急性疼痛控制不佳容易诱发慢性疼痛，后者严重影响患者术后远期生活质量^[12]^。患者术后疼痛的减轻有助于患者术后咳嗽排痰，并增加患者进行术后早期康复活动的依从性。因此，综合应用各种手段控制患者术后疼痛是提高微创手术效果的关键因素。本研究发现，单孔组术后24 h NRS评分和72 h NRS评分均优于三孔组，结果有统计学差异。Tamura等^[13]^回顾性分析了19例单孔胸腔镜手术和18例三孔胸腔镜手术患者的临床资料后发现，三孔组在术后24 h VAS疼痛评分方面差于单孔组，有统计学差异。贾斌等^[14]^在术后24 h VAS评分方面研究发现，83例单孔组患者在疼痛程度上低于439例三孔组患者，比较有统计学差异。其主要原因在于，单孔手术最大限度地减少了肋间神经的损伤，另外，软性切口保护套的使用避免了戳卡和副操作孔器械使用对切口反复挤压和摩擦导致的术后切口疼痛。

在完成学习曲线后，绝大多数术者能够在不增加手术时间和中转率的前提下完成单孔胸腔镜肺叶切除，已被学术界所接受。但单孔胸腔镜是否能完成同样的淋巴结清扫范围，文献报道较少。系统性肺门纵隔淋巴结清扫是肺癌手术重要环节之一，淋巴结清扫的范围对肺癌患者术后分期和指导后续辅助治疗至关重要。NCCN的NSCLC治疗指南及欧洲胸外科医师学会均指出行可切除NSCLC手术时应行系统性肺门纵隔淋巴结清扫，且N2淋巴结群切除不少于3站。本研究中，单孔组淋巴结清扫站数和枚数与三孔组比较无统计学差异。Shao等^[15]^通过对48例行单孔和52例行三孔胸腔镜手术的Ⅰ期和Ⅱ期肺癌患者的临床资料进行分析后发现，单孔组淋巴结清扫站数和数目分别为7.5和12.8，三孔组淋巴结清扫站数和数目分别为7.1和13.6，两组在淋巴结清扫站数和数目上无统计学差异。Shen等^[16]^通过对115例行单孔VATS和296例行三孔VATS的肺癌患者进行倾向性匹配分析发现，单孔组和三孔组淋巴结清扫数分别为21.4和20.9，无统计学差异。我们认为，术中器械的合理摆放和正确的局部暴露是单孔胸腔镜淋巴结清扫的关键。对此，我们的经验是：胸腔镜镜头始终位于切口的上缘，用于牵拉暴露的圈钳纱布位于切口的下缘，主刀在切口中央使用能量器械和吸引器，基本可以完成所有位置的淋巴结清扫。另外，在清扫淋巴结过程中，尽量沿淋巴结外膜完整切除，区域淋巴结组尽量做到整群切除，减少术中出血影响手术视野。

相对于传统开放手术，胸腔镜肺部手术显著改善了手术的创伤，减少了术后并发症的发生率，提高了患者术后远期和近期生活质量^[17, 18]^。文献报道，胸腔镜手术在切口感染，术后心脏和肺部并发症方面明显优于传统开放手术。本研究发现，单孔组术后并发症发生率较三孔组为低，但结果无统计学差异。French等^[19]^研究50例行单孔VATS肺癌患者和50例行三孔VATS肺癌患者发现，单孔组术后微小并发症发生率为18%，三孔组术后微小并发症发生率为26%，两组在术后并发症上无统计学差异。Mcelnay等^[20]^对比分析了15例行单孔胸腔镜手术和95例行多孔胸腔镜手术的术后并发症情况，发现在术后并发症发生率上两组无统计学差异。我们认为其主要原因在于：①更少的手术切口减少了术后切口疼痛，有利于患者术后有效咳嗽排痰，促进了肺的复张和患者的氧合状态；②单孔手术由于切口的减少本身降低了切口感染的可能性；③更大限度地保持了患者的胸壁的完整性，有助于患者术后的胸式呼吸恢复；④以上研究均发现单孔组并发症发生率低于三孔组并发症发生率，但均无统计学差异，可能在于例数均较少。

本研究也存在一些不足之处：①本研究为单中心回顾性研究，存在一定的病例选择性偏倚；②虽然两组患者一般临床资料比较无统计学差异，具有可比性，但可能医生和患者主观选择更简单的手术，接受了单孔手术，且手术解剖也更为简单，出血量也必然减少，术后恢复也更快。其结果有待更大样本量的前瞻性对照研究进一步证实。

综上所述，单孔VATS作为一种更加微创的手术，治疗可切除肺癌安全有效，其能够达到三孔VATS同样的肿瘤学切除范围，并在术中出血量、术后疼痛、术后引流量、术后胸管留置时间和术后住院时间方面优于三孔VATS肺部手术。因此，在病例合适的情况下，应该优先选择单孔VATS，这样有利于患者的术后恢复，有助于提高患者术后生活质量。
